# Identification of key player genes in gene regulatory networks

**DOI:** 10.1186/s12918-016-0329-5

**Published:** 2016-09-06

**Authors:** Maryam Nazarieh, Andreas Wiese, Thorsten Will, Mohamed Hamed, Volkhard Helms

**Affiliations:** 1 Center for Bioinformatics, Saarland University, Saarbruecken, Germany; 2 Graduate School of Computer Science, Saarland University, Saarbruecken, Germany; 3 Max Planck Institut fuer Informatik (MPII), Saarbruecken, Germany; 4 Institute for Biostatistics and Informatics in Medicine and Ageing Research, University of Rostock, Rostock, Germany

**Keywords:** Minimum dominating set, Minimum connected dominating set, Gene regulatory network, Integer linear programming, Heuristic algorithm

## Abstract

**Background:**

Identifying the gene regulatory networks governing the workings and identity of cells is one of the main challenges in understanding processes such as cellular differentiation, reprogramming or cancerogenesis. One particular challenge is to identify the main drivers and master regulatory genes that control such cell fate transitions. In this work, we reformulate this problem as the optimization problems of computing a Minimum Dominating Set and a Minimum Connected Dominating Set for directed graphs.

**Results:**

Both MDS and MCDS are applied to the well-studied gene regulatory networks of the model organisms *E. coli* and *S. cerevisiae* and to a pluripotency network for mouse embryonic stem cells. The results show that MCDS can capture most of the known key player genes identified so far in the model organisms. Moreover, this method suggests an additional small set of transcription factors as novel key players for governing the cell-specific gene regulatory network which can also be investigated with regard to diseases. To this aim, we investigated the ability of MCDS to define key drivers in breast cancer. The method identified many known drug targets as members of the MDS and MCDS.

**Conclusions:**

This paper proposes a new method to identify key player genes in gene regulatory networks. The Java implementation of the heuristic algorithm explained in this paper is available as a Cytoscape plugin at http://apps.cytoscape.org/apps/mcds. The SageMath programs for solving integer linear programming formulations used in the paper are available at https://github.com/maryamNazarieh/KeyRegulatoryGenesand as supplementary material.

**Electronic supplementary material:**

The online version of this article (doi:10.1186/s12918-016-0329-5) contains supplementary material, which is available to authorized users.

## Background

Although all the cells in multicellular organisms basically share the same DNA sequence with the same set of genes, in each cell type only a particular set of genes is actively expressed which then defines its specific morphology and function. Thus, different types of cells are controlled by different sets of active genes and by the interactions between them [1–4]. Inside each cell, a set of target genes and regulatory genes, namely the transcription factors (TFs), interacts with each other and forms a gene regulatory network (GRN). GRNs topologically comprise a highly connected component and a few nodes with low connectivity [5]. Embryonic stem cells (ESCs), for example, can be distinguished from other cells mainly based on their pluripotency network. This network in ESCs is spanned up by few connected TFs which share many target genes [6]. A slight change in the expression levels of such a tightly interwoven network of TFs leads to ESC differentiation [6].

Of particular interest are the groups of key driver genes and master regulatory genes in condition-specific and unspecific gene regulatory networks. Key driver genes are basically those genes that control the state of the network [7–9]. The term master regulatory gene was introduced by Susumu Ohno over 30 years ago. According to his definition, a master regulator is a gene which stands at the top of a regulatory hierarchy and is not regulated by any other gene [10]. Later on, this term was redefined to involve a set of genes which either directly govern the particular cellular identity or are at the inception of developmental lineages and regulate a cascade of gene expressions to form specific lineages [11].

To address the problem of computational identification of key and master regulatory genes, we have modeled and solved two optimization problems named Minimum Dominating Set (MDS) and Minimum Connected Dominating Set (MCDS) on the GRNs. We compared these sets against well-known centrality measures such as degree, betweenness and closeness centrality as described in [12]. These attribute the importance of genes to their centrality in the networks. However, it is unclear whether high centrality genes provide a full control of the underlying network.

A recent study derived a minimum input theorem based on structural control theory which can be applied to directed graphs to fully control the network [7]. For this, the authors introduced a deep relation between structural controllability and maximum matching. The idea is to control the whole network by covering all the regulatory interactions with a minimum number of genes. Their results show that a few nodes are sufficient to control dense and homogeneous networks, but this number increases dramatically when the nodes in the network are sparsely connected.

**Fig. 1:**
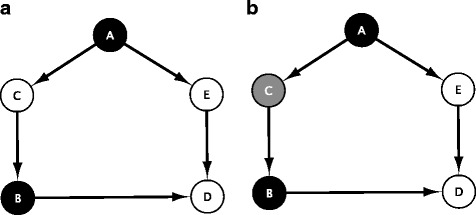
A graphical representation that illustrates the MDS and MCDS solutions of an example network. The network can be controlled by MDS and MCDS nodes. In the case of a GRN, directed arcs symbolize that a transcription factor regulates a target gene. In panel **a**, the MDS nodes {*A,B*} are the dominators of the network. Together, they regulate all other nodes of the network (*C, E, D*). Panel **b** visualizes the respective set of MCDS nodes (*black and gray*). Here, node *C* is added in order to preserve the connection between the two dominators *A* and *B* to form an MCDS

An MDS is a related concept in which the goal is to control the network by covering all expressed genes with a minimum number of TFs. Since each node that does not belong to the MDS is adjacent to at least one node in the MDS, full control over the network is provided by the MDS solution. Our group has previously applied the concept of MDS to the area of complex diseases. The results showed that this method can capture several important disease and drug target genes [9, 13]. The MDS method can be applied to any connected or disconnected regulatory network to identify key dominator nodes. In this work, we use MDS in directed graphs to identify key driver genes. Besides the MDS concept, we suggest to also consider the task of identifying a set of master regulatory genes as an analogue of another optimization problem, namely that of constructing an MCDS. We suggest to apply MCDS mainly to networks that are related to cell fate transitions such as the pluripotency network of an embryonic stem cell. This idea is motivated by the observation that the pluripotency network in mouse ESCs is maintained by a few connected TFs which share many target genes [6]. The concepts of MDS and MCDS are visualized for a small toy network in Fig. 1.

The concept of MCDS has already been applied to protein-protein interaction networks (which are represented by undirected graphs). There, the proteins which compose a MCDS solution contributed significantly to related biological processes [14]. In this work, we show how the MCDS concept can be applied to GRNs (represented by directed graphs) to detect the TFs and target genes which determine a specific cellular identity. We start with the model organisms *E. coli* and *S. cerevisiae* because their GRNs have been extensively characterized in experimental studies. Then, we present applications to a mouse pluripotency network and to a breast cancer regulatory network.

## Methods

### Minimum dominating set

A *dominating set* (DS) in an undirected graph *G*=(*V*,*E*) is a subset of nodes *D*⊆*V* with the property that for each node *v*∈*V* we have that *v*∈*D* or that there is a node *u*∈*D* and an edge {*u*,*v*}∈*E*. We call a set *D*⊆*V* a *minimum dominating set* (MDS) if it is a dominating set and it has minimum cardinality among all dominating sets for *G*. Computing a MDS is known to be an NP-complete problem [15]. In biological networks, the set of dominators can provide full control over the whole network. Since each node that does not belong to the MDS is at least adjacent to one node in the MDS, full control over the network can be obtained by the MDS solution. To address GRNs which are represented by directed graphs, we define an MDS for a directed graph *G*=(*V*,*E*) to be a set *D*⊆*V* of minimum cardinality such that for each node *v*∈*V* we have that *v*∈*D* or that there is a node *u*∈*D* and an arc (*u*,*v*)∈*E*. The integer linear programming (ILP) formulation of MDS for directed graphs is given below. Here, for each node *v*∈*V* we denote by *δ*^−^(*v*) the set of incoming nodes of *v*, i.e., the set of nodes *u* such that (*u*,*v*)∈*E*.

1 $$ \begin{aligned} & {\text{minimize}} && \sum_{v\in V} x_{v}\\ & \text{subject to} & & x_{u} + \sum\limits_{v \in \delta^{-}(u)} x_{v} \geq 1 & \forall u\in V\\ && & x_{v} \in \{0,1\} & \forall v\in V \end{aligned}  $$

Here, variables *x*_*u*_ and *x*_*v*_ are binary variables associated to the nodes *u* and *v* in the graph. Using this formulation, we select a node *v* as a dominator if its binary variable *x*_*v*_ has value 1 in the computed solution and otherwise we do not select it. Since our objective function is to minimize $\sum _{v\in V} x_{v}$ this yields a minimum dominating set. For all networks considered here, MDS solutions were constructed in less than 1 minute of running time.

### Minimum connected dominating set

A minimum connected dominating set (MCDS) for a directed graph *G*=(*V*,*E*) is a set of nodes *D*⊆*V* of minimum cardinality that is a dominating set and that additionally has the property that the graph *G*[*D*] induced by *D* is weakly connected, i.e., such that in the underlying undirected graph between any two nodes *v*,*v*^′^∈*D* there is a path using only vertices in *D*. Computing an optimal MCDS in undirected graphs is known to be NP-hard [15]. Since GRNs are represented by directed graphs, we are interested in MCDSs for directed graphs.

#### Optimal solution via ILP

To this end, we modified the existing integer linear programming (ILP) formulation of MCDS in undirected graphs [16] to determine a MCDS for directed graphs.

As before, the set *V* is the set of vertices and *E* is the set of edges in the input graph. For a set *S*⊆*V*, the set *E*(*S*) stands for all the edges connecting two vertices *u*,*v* with *u*,*v*∈*S*. The binary valued *y*_*v*_ variables indicate whether node *v* is selected to belong to the minimum connected dominating set. The binary variables *x*_*e*_ for the edges then yield a tree that contains all selected vertices and no vertex that was not selected. Thus, the selected vertices form a connected component. The first constraint guarantees that the number of edges is one unit less than the number of nodes. This is necessary for them to form a (spanning) tree but is not sufficient. The second constraint guarantees that the selected edges imply a tree. The third constraint guarantees that the set of selected nodes in the solution forms a dominating set of the graph. For dense undirected graphs, this formulation provides a quick solution, but in the case of sparse graphs, finding the optimal solution may take considerable running time [16].

2 $$ \begin{aligned} &{\text{minimize}} && \sum \limits_{v \in V}y_{v} \\ & \text{subject to} & & \sum\limits_{e \in E} x_{e} = \sum\limits_{i \in V} y_{i} -1\\ & & & \sum\limits_{e \in E(S)} x_{e} \leq \sum\limits_{i \in S\setminus \{j\}}y_{i} & \forall S \subset V, \forall j \in S \\ && & y_{u} + \sum\limits_{v \in \delta^{-}(u)} y_{v} \geq 1 & \forall u\in V \\ & && y_{v} \in \{0,1\} & \forall v\in V \\ & && x_{e} \in \{0,1\} & \forall e\in E & \end{aligned}  $$

The above IP formulation contains an exponential number of constraints since it has one constraint for each subset *S*⊆*V*. Therefore, already for relatively small instances it is impractical to generate all its inequalities. Instead, we used the following approach: we generate the first constraint and all constraints of the third type (i.e., $\sum \limits _{e \in E} x_{e} = \sum \limits _{i \in V} y_{i} -1$ and $y_{u} + \sum \limits _{v \in \delta ^{-}(u)} y_{v} \geq 1 $ for each *u*∈*V*). Then we compute the optimal IP solution subject to these constraints. Then we check whether the found solution satisfies *all* constraints of the above IP (even those that we did not add to our formulation). This is the case if and only if the computed set of vertices yields a connected (dominating) set. If this is the case then we found the optimal solution and we stop. Otherwise, we add (violated) constraints of the second type (i.e., $\sum \limits _{e \in E(S)} x_{e} \leq \sum \limits _{i \in S\setminus \{j\}}y_{i}$ for some subset *V* and some node *j*) to our formulation and compute the optimal IP solution to this stronger formulation and repeat. If the computed set of vertices has more than one connected component then we add such a constraint for each connected component *S* and for each vertex *j*∈*S*. In order to improve the running time of our procedure, we added some valid inequalities to our initial formulation. These inequalities discard all the solutions that select an edge *e*={*u*,*v*} (i.e., *x*_*e*_=1) such that not both of its incident vertices were selected (i.e., not both *y*_*u*_=1 and *y*_*v*_=1). Formally, for each edge *e*={*u*,*v*} we added the inequalities

3 $$ \begin{aligned} x_{e} \leq y_{u}\\ x_{e} \leq y_{v} \end{aligned}  $$

Despite adding these valid inequalities, some problem instances were not solved in appropriate time. To overcome this problem, we also considered a heuristic approach. It is known that an approximate MCDS can be found by heuristic approaches in polynomial time [17, 18]. For graphs with low node density and high node degree, the optimal ILP solution can be found at comparable running times as such heuristic solutions [17]. However, the heuristic solution outperforms the ILP for graphs with high node density and low node degree in terms of running time [17]. In this work, all computations were conducted on a single threaded Intel XEON CPU at 2.2 Ghz. We determine the ILP solution using the glpk solver version 4.35 [19]. In cases where the network is very sparse we used the heuristic algorithm (see next section).

#### Heuristic solution

In this study, we computed the heuristic solution for all networks except for the modules of a breast cancer network. There, the optimal MCDS solution could be obtained within a few minutes to several hours of compute time. We adapted the heuristic algorithm presented in [18] that was inspired by one of the two general approximation approaches mentioned in [20] to find solutions for MCDS. We modified the algorithm to determine a MCDS for directed graphs rather than an undirected graph. The algorithm has three main phases as described in the following. Initially, all nodes are white. In the first phase, a white node with the highest outdegree is selected as a dominator and colored black. In cases where multiple nodes have the same outdegree, we select the node with the highest indegree. This selection guarantees higher connectivity compared to nodes with smaller indegree. Its (directed) child neighbors are colored gray to indicate that they are already dominated. This step is repeated until all nodes are either black or gray. From these, we check if the (black) set of dominators forms a connected dominating set. If yes, we move to the third phase, otherwise we move to the second phase. In the second phase, a node with maximum number of arcs to black nodes, that we term a connector, is colored dark gray. This dark gray node is then added to the connected dominating set if it belongs to a path between two connected components that are not connected so far. This step is repeated until all black and dark gray nodes form a connected component in the underlying undirected graph. In the third phase, the size of the connected dominating set is reduced as much as possible by repeatedly removing a node with smallest outdegree while making sure that the dominating set remains connected and the graph remains covered by the connected dominating set. In cases where multiple nodes have the same outdegree, we again select the node with highest indegree.

One can also interpret the algorithm biologically in the context of GRNs. We start by selecting a TF with the most target genes as a dominator. This process is repeated until all the genes are either selected as dominators or as target genes. If the dominating set is not connected, the next step is to connect the dominators by adding a few number of connector genes. This step is motivated by the modularity of cellular networks [21]. We will investigate below whether defining a connected set of dominator nodes is beneficial for the biological interpretability of the control hierarchy. As connectors, we consider TFs as well as target genes. The last step is to reduce the size of the connected dominating set. Then, the connected dominating set comprises of dominators and connectors, whereby all dominators are TFs and the connectors comprise of TFs and/or target genes. Note that the set of MCDS identified as dominators or connectors provides potential candidates for key drivers and master regulatory genes.

For the networks considered here, the running time for the heuristic MCDS solution was less than 1 minute.

### Components

Unlike MDS, the task of computing an MCDS only makes sense for input graphs that are connected since otherwise there can be no solution. Therefore, if we are given a disconnected undirected graph, we compute MCDSs for connected components of the graph. For directed graphs, we distinguish between strongly connected components and (weakly) connected components.

#### Strongly connected component

A component is called a strongly connected component (SCC) in a directed graph if each of its nodes is reachable via directed edges from every other node in the component. In a SCC, there is a path between each pair of nodes in the component. Here, we implemented Tarjan’s algorithm to find SCCs as described in [22].

#### Largest connected component

**Fig. 2:**
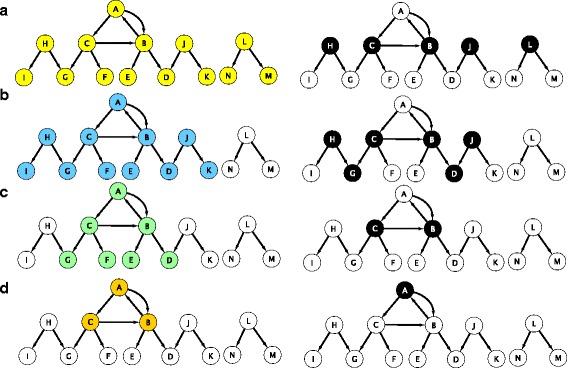
A graphical representation that illustrates the concept of MDS on a toy network. In addition, the MCDS nodes are colored black on three types of components (LSCC, LCC of the underlying directed graph and LCC of the underlying undirected graph) in the toy network. The above toy network includes 14 nodes and 14 edges as shown in *yellow* in panel (**a**). The nodes {*J, B, C, H, L*} are the dominators of the network obtained by computing a MDS (*right panel*). The *nodes colored blue* in panel **b**, make up the largest connected component of the underlying undirected graph. MCDS nodes for this component are {*J, D, B, C, G, H*}. *Green colored nodes* in panel **c** are elements of the largest connected component underlying the directed graph. The two nodes {*B, C*} form the MCDS for this component. The *nodes colored orange* in panel **d** show the LSCC in the network. Here, the node *A* is the only element of the MCDS

A component is a (weakly) connected component if in the underlying undirected graph, there exists a path between any pair of nodes of this component. The connected component of highest cardinality is termed the largest connected component (LCC). The connected components were found by breadth first search (BFS) as described in [23]. Note that each strongly connected component is also a (weakly) connected component but the converse is not necessarily true. Since a MCDS does not exist in graphs that are not connected, we consider the largest connected component (LCC) and the largest strongly connected component (LSCC) in such cases, see Fig. 2. We compared the results of MCDS when the network has only one connected component to those obtained with a directed version of MDS in terms of the size of the result set and enrichment analysis.

#### Criteria to select the component

MDS is always applied to the whole network. If the input network is not connected, we select either LCC or the LSCC as the input for MCDS. If the cardinality of the network is equal to the LCC of the network, we select the whole network. Otherwise, we consider the component density of LCC and LSCC. For a directed graph *G*=(*V*,*E*), the component density is defined as $\frac {|E|}{|V| (|V| - 1)}$, where *E* denotes the set of edges and *V* denotes the set of nodes in the component. The component density is equal to the ratio of existing edges (interactions) |*E*| in the component to the total number of possible edges (interactions). According to the definitions in [24], in a dense graph the number of edges is close to the maximal number of edges which is in contrast to a sparse graph. In this study, an MCDS is then derived for the component (LCC or LSCC) with highest density, as we were interested to find the minimum number of genes. High density components are more promising in this regard, because they need a smaller number of connectors to connect the dominators.

### Enrichment analysis

The biological relevance of the results obtained by the directed forms of MDS and MCDS was evaluated using the enrichment analysis tool provided at the DAVID portal of NIH [25]. *p*-values below the threshold 0.05 obtained by the hypergeometric test were adjusted for multiple testing using the Benjamini & Hochberg (BH) procedure [26].

### Functional similarity

Functional similarity was examined based on Gene Ontology (GO) Biological Process (BP) terms among the pairs of MCDS nodes. This was then compared to the functional similarity of gene pairs from the entire network as described in [27]. The permutation test was repeated 100 times and Kolmogorov-Smirnov (KS) test was applied to get the *p*-value.

### Hypergeometric test

The statistical significance of the results was assessed using the hypergeometric test which is based on sampling without replacement. The *p*-value for the test is calculated from the following formula:

$$p-value = 1 - \sum_{i = 0}^{x}\frac{{k \choose i}{M-k \choose N-i}}{{M\choose N}}$$

where *M* is the total number of genes in the network, *N* is the sample size which is equal e.g. to the size of the MCDS, *k* is the number of genes in *M* with a specific property and *x* is the number of genes in the MCDS having that property. The cutoff value was set to *p*=0.05 to report a set obtained by MCDS as a significant result. To apply the test, we used the online tool (GeneProf) which is described in [28].

### Data and software

We tested the presented approaches to identify key player and master regulatory genes in several GRNs for *E. coli*, *S. cerevisiae*, a human breast cancer network and for the pluripotency of mouse ESC. We will present the obtained results one by one in the next section.

The dataset of *E. coli* is a GRN of the *Escherichia coli* strain K-12 that was downloaded on 22-July-2014 from RegulonDB [29]. It contains curated data for 1807 genes, including 202 TFs.

The dataset of *S. cerevisiae* was taken from the Yeast Promoter Atlas (YPA) downloaded on 26-March-2014 [30]. It contains 5026 genes including 122 TFs. In this database, the target genes for each TF is a set of genes whose promoter regions contain the associated transcription factor binding site for the TF binding motif.

The dataset for mouse is a manually curated GRN of mouse (*Mus musculus*) ESCs. It consists of 274 mouse genes/proteins and 574 molecular interactions, stimulations and inhibitions [31]. The network consists of genes that are involved in either induction, maintenance or loss of the pluripotency state and is thus termed pluripotency network throughout the text.

The breast cancer network used here was generated in [9] using a Bayesian learning approach that was coupled to an integrative network-based approach based on whole-genome gene expression profiling, DNA methylome, and genomic mutations of breast cancer samples from TCGA. The GRN networks were constructed via three steps: first the co-expression network was generated based on the topological overlap matrix as a distance measure. Then, we connected the co-expression interactions to regulatory information retrieved from publicly available regulatory databases accompanied with motif search for all known binding motifs of the TFs represented in the co-expression network against the promoter regions of all genes in the network. Finally, a causal probabilistic Bayesian network was inferred from the co-expression modules utilizing the directed edges obtained from the previous step as a start search point to infer directionality between nodes. Clustering yielded ten network modules of dysregulated genes [9]. Each module turned out to have distinct functional categories, cellular pathways, as well as oncogene and tumor suppressor specificity. We also extracted breast cancer specific subnetworks from the human genome regulatory interactome induced by the dysregulated mRNAs.

We implemented the ILP formulas for the directed forms of MDS and MCDS in the SageMath software system [32] version 6.8 using the glpk solver [19]. We implemented the heuristic algorithm in Java and made it available as a plugin for the popular biological network analysis platform Cytoscape [33] at http://apps.cytoscape.org/apps/mcds and in Additional files 1 and 2. Additional file 3 provides a user guide and example files. Additional file 4 provides the GRN Networks used in this study.

## Results and discussion

### Global *E. coli* GRN

The GRN for *E. coli* studied here contains 1807 genes, including 202 TFs and 4061 regulatory interactions. This set of regulatory interactions in *E. coli* forms a general network which controls all sorts of responses which are needed in different conditions. With network density 0.001, the network can be considered as sparse. Due to this sparsity, MDS deems 199 TFs to be necessary to control the network. The network does not have any SCC with size larger than 5 nodes. For computing an MCDS, we therefore used the LCC underlying directed graph that contains 1198 genes. Based on the directed form of the LCC, target genes are placed at the bottom level and a set of TFs comprises the MCDS. In the LCC, the algorithm identified an heuristic MCDS containing 34 genes (11 dominators and 23 connectors) that cover the entire component, see Additional file 5: Table S1.

Additional file 5: Figure S1 illustrates the hierarchical structure between the 34 TFs contained in the MCDS. The hierarchical structure was drawn based on generalized hierarchies using breadth-first search as described in [34]. A previous study that was based on an earlier version of RegulonDB identified 10 global regulators that regulate operons in at least three modules [35]. Two of them, H-NS and CspA, do not belong to the LCC considered here. Two other global TFs identified previously (RpoS and RpoN) are no longer contained in the list of regulators in the version of RegulonDB used here. Out of the six remaining genes, the five genes IHF, CRP, FNR, ArcA and NarL are among the nine top genes in Table S1 and the sixth gene OmpR is found a bit further below in the list. Table S2 lists enriched KEGG and GO terms for the 34 genes in the MCDS of the *E. coli* gene regulatory network. As expected, the strongest enrichment is found for processes related to transcriptional regulation. The second most enriched term is related to two-component systems which enables *E. coli* to respond to changes arising from different environmental conditions [36].

### Cell-cycle specific *S. cerevisiae* GRN

**Fig. 3:**
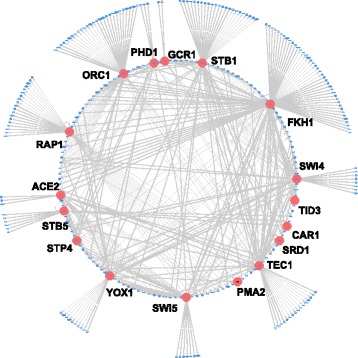
Tightly interwoven network of 17 TFs and target genes that organize the cell cycle of *S. cerevisiae*. Shown on the circumference of the outer circle are 164 target genes that are differentially expressed during the cell cycle. The inner circle consists of the 14 TFs from the heuristic MCDS and of 123 other target genes that are regulated by at least two of these TFs

Next, we retrieved regulatory interactions in *S. cerevisiae* involving 122 TFs from the Yeast Promoter Atlas (YPA) [30]. From this set of regulatory interactions, we extracted a cell-cycle specific subnetwork of 302 genes that are differentially expressed along the cell cycle of yeast as described in [37]. The 302 genes already form the LCC of this subnetwork. This set of genes is controlled by a MDS including 12 TFs and a heuristic MCDS including 14 TFs and 3 target genes. The MDS and MCDS elements are listed in Tables S3 and S4, respectively. Most of the TFs identified to belong to the MDS and MCDS have been identified before by experimental methods to be associated with the cell cycle [38]. Figure 3 shows the GRN of the cell cycle activity of *S. cerevisiae* controlled by these 14 TFs. Table S5 lists enriched KEGG and GO terms for the 17 genes in this MCDS. As expected and similar to what we found for the *E. coli* network, the strongest enrichment was found for processes related to transcriptional regulation. 9 of the 17 genes (PMA2, YOX1, ACE2, SWI5, SWI4, ORC1, STB1, FKH1, TID3) are annotated to cell-cycle related GO terms, namely GO:0051329 ∼ interphase of mitotic cell cycle and GO:0000278 ∼ mitotic cell cycle and to the KEGG pathway sce04111:Cell cycle.

### Pluripotency network in mouse

**Fig. 4:**
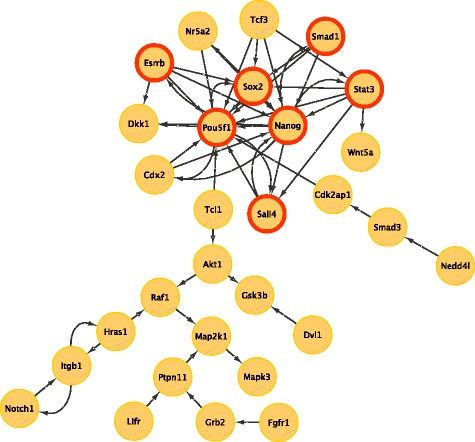
Connectivity among TFs in the heuristic MCDS of the largest strongly connected component of a GRN for mouse ESCs. The red circle borders mark the 7 TFs belonging to the set of master regulatory genes identified experimentally in [39]

Next, we applied the MDS and MCDS methods to a manually curated GRN of mouse ESCs that consists of 274 mouse genes/proteins and 574 molecular interactions, stimulations and inhibitions [31]. We found that the heuristic MCDS of the LSCC (80 genes) of this network contains 29 TFs. The connectivity among these 29 TFs is displayed in Fig. 4. The MCDS elements are listed in Table S6, respectively. Among the set of regulators, 7 TFs including *Pou5f1, Nanog, Sox2, Stat3, Esrrb, Tcf3, Sall4* are in common with an experimentally validated regulatory network controlling pluripotency that consists of 15 experimentally validated TFs [39]. Such a result is unlikely to be obtained by chance (hypergeometric test *p*-value = 0.004) in a network with 176 TFs.

Next, we evaluated the ability of the MCDS method to detect a cooperative biologically functional backbone within the entire network. For this, we examined the functional similarity according to the Wang measure in the GoSemSim R package [40], among the pairs of MCDS nodes and compared this to the functional similarity of gene pairs from the mouse network, see Additional file 5: Figure S2. This figure shows the cumulative distribution of the functional similarity scores between pairs of MCDS nodes of the mouse pluripotency network (in red) compared to the similarity scores of all possible pairs between genes of this network (in black). The Kolmogorov-Smirnov test revealed that the MCDS genes were functionally significantly more homogeneous than the randomly selected gene pairs of the whole network with *p*-value of 6.41e-05. This hints at the ability of the MCDS method to extract a functionally homogeneous network backbone that is expected to have an important role in maintaining the pluripotency state in early developmental stages. Table S7 lists enriched KEGG and GO terms for the 29 genes in the MCDS of the mouse ESC pluripotency network. In this case, GO terms related to developmental processes are stronger enriched than GO terms related to transcriptional regulation. The set of genes (Nanog, Cdx2, Esrrb, Pou5f1, Sox2 and TCl1) annotated with GO:0019827 are responsible for stem cell maintenance. The genes annotated with other GO terms are mainly related to embryonic development and other tissue-specific development.

**Fig. 5:**
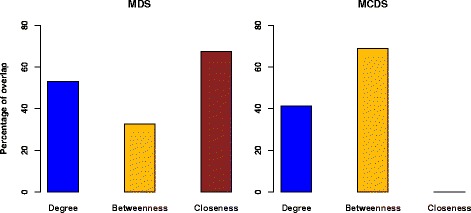
Percentage overlap of the genes of the MDS and MCDS with the list of top genes (same size as MCDS) according to 3 centrality measures. Shown is the percentage of genes in the MDS or MCDS that also belong to the list of top genes with respect to degree, betweenness and closeness centrality

To check the centrality significance of the MCDS genes in the LSCC, we selected the same number of genes as the size of MCDS with respect to degree, betweenness and closeness centrality. The centralities were measured using the igraph package [41]. We considered only outdegree nodes in the directed network. The results show that most of the genes contained in the heuristic MCDS are among the top nodes according to at least one centrality (degree, betweenness, closeness), see Fig. 5. Among them, the top nodes of the MCDS have the highest overlap with the top nodes of the degree centrality and the betweenness centrality. Six out of 10 connector nodes in MCDS belong to the top 29 nodes with highest betweenness centrality according to Jaccard’s index.

### Human disease network

Finally, we applied the MCDS method to the LCC of ten breast cancer network modules where each LCC covers the whole module [9], see “Methods” section.

Table 1 lists the identified MDS and MCDS sets for the nine out of ten modules. One module (grey) could not be solved in appropriate time using ILP. In total, the MDS and MCDS sets of the nine modules contain 68 and 70 genes, respectively. Then, we looked up the known anti-cancer drugs that target any of the 70 proteins coded for by these genes based on experimentally validated drug-target databases as described in [9]. In the network with 1169 genes including 228 drug target genes, we found that 20 of the 70 drug target genes belong to the genes identified using the MCDS. This is statistically significant with a *p*-value of 0.03 obtained from the hypergeometric test. Sixteen out of the 68 proteins belonging to the MDS genes are binding targets of at least one anti-breast cancer drug, see Table 1.

**Table 1:** Identified genes in the MDS and MCDS (ILP) for 10 modules of the breast cancer network

Method	Module	Network size	Result size	Key driver genes
MCDS	Black	41	5	ZNF254, KIAA1632, ZNF681, SEC24B, ZNF615
MDS			5	ZNF254, KIAA1632, ZNF681, SEC24B, ZNF615
MCDS	Blue	247	3	FAM54A,**ACAN**, GLDC
MDS			2	**ACAN**, FAM54A
MCDS	Brown	195	1	**AATK**
MDS			1	**AATK**
MCDS	Green	110	18	**ADPRHL2**, **AKT1**, LTBR, MAN2C1, SH3GLB2, UTP14A, WDR55, MADD, **B4GALT7**, **OS9**, MYO1C, **CDC34**, **CDC37**, RBM19, **MARS**, **CCDC22**, MAP2K2, DAP
MDS			17	**ADPRHL2**, LTBR, HMG20B, **HK1**, SH3GLB2, UTP14A, ELK1, MED6, **B4GALT7**, **OS9**, MYO1C, **CDC34**, CLN3, **INPPL1**, DAP, PLXNB1, TIMM44
MCDS	Magenta	26	4	ILF2, **BGLAP**, POGK, **ATF6**
MDS			4	ILF2, **BGLAP**, **ATF6**, VPS72
MCDS	Pink	30	5	TCEB1, RAB2A, ZNF706, TMEM70, **ATP6V1C1**
MDS			5	TCEB1, RAB2A, TMEM70, TCEA1, **ATP6V1C1**
MCDS	Red	93	13	SIX4, **SP1**, **ATP1B1**, PCGF1, SUMF2, EPN3, GTF3A, RAP1B, FHL3, RPS3A, **ABCB8**, GFAP, **ANXA5**
MDS			13	LSM11, SIX4, PCGF1, SUMF2, EPN3, ZNRF2, GTF3A, RAP1B, FHL3, RPS3A, **ABCB8**, GFAP, NAGA
MCDS	Tur quoise	295	1	**ABHD10**
MDS			1	**ABHD10**
MCDS	Yellow	132	20	**CASP10**, TSPAN2, **ACSL6**, HDAC11, SLC7A7, TRAF3IP3, GZMK, PAG1, LAP3, HTRA4, **CD79B**, SPI1, GCET2, WAS, DFNA5, LRRC33, FCRL2, LCP2, TCTEX1D1, FUT4
MDS			20	**CASP10**, TSPAN2, **ACSL6**, HDAC11, TLR9, SLC7A7, FAM129C, TRAF3IP3, HTRA4,SPI1, CPXM2, GCET2, **FASN**, SLFN11, DFNA5, ETS1, PLS3, LCP2, TCTEX1D1, FUT4

The genes, whose protein products are known to be targeted by drugs, are marked in bold

**Fig. 6:**
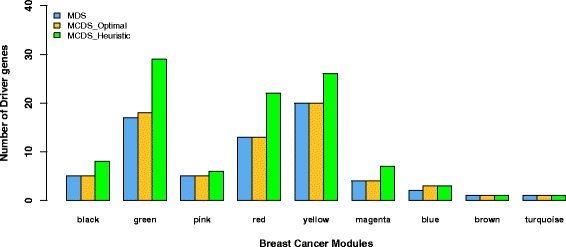
Number of MCDS genes determined by the heuristic approach or by the ILP formulation and in the MDS. Shown are the results for 9 modules of the breast cancer network

Next, we compared the set size of the optimal and heuristic solutions of MCDS and MDS for 9 out of the 10 modules. One module (grey) could not be solved in appropriate time using ILP. Table S8 displays the density and running time for the ILP solutions for the mentioned modules implemented in Sage. The running time was not correlated with the size or density of the networks. Figure 6 shows that the optimal solutions of MCDS and MDS contain almost the same number of genes for all modules. In comparison, the heuristic MCDS solutions (see, Table S9) contain 10–50 % more genes than the solutions of the other two approaches. We also compared the heuristic approach with the optimal solution in terms of overlapped identified genes. Table 2 indicates that according to Jaccard’s index the solutions overlap approximately by about 60 % in a range from 40 to 75 %. Table S9 shows the results obtained by the heuristic approach of MCDS. Table S10 lists enriched GO BP terms and KEGG pathways in the MCDS genes obtained by heuristic approach. 12 genes (AKT1, RASSF5, WNT5B, ETS1, PDGFA, TP53, SPI1, NFKB1, TCEB1, MYC, TGFB1, DAPK1) belong to a known cancer pathway (*p*-value = 0.004). We hypothesize that the products of some of the remaining identified MCDS protein coding genes may open up new avenues for novel therapeutic drugs.

**Table 2:** Overlapping genes between the heuristic and optimal solutions of MCDS for modules of the breast cancer network. The names of the modules were introduced in the original ref. [9]

Module	Shared genes	Count
black	SEC24B, ZNF254, ZNF681	3
green	UTP14A, LTBR, SH3GLB2,	
	OS9, CDC34, CDC37, AKT1	7
magenta	BGLAP, ATF6, ILF2	3
pink	ZNF706, TCEB1, TMEM70	3
red	FHL3, SUMF2, RPS3A, PCGF1,	
	EPN3, GTF3A, ATP1B1	7
yellow	FUT4, SPI1, DFNA5, CASP10, PAG1,	
	HDAC11, LCP2, TRAF3IP3, HTRA4, TSPAN2, GZMK	9
blue	ACAN, FAM54A	2

The modules brown and turquoise have only 1 mcds gene and give 100 % overlap

### Directed random networks

To characterize the size of problems which can be solved using the MCDS ILP formulation, multiple Erdos-Renyi random digraphs were generated using the Java code DigraphGenerator available in [42] with different sizes and densities. We discarded the networks whose running times exceeded 2 days. Table S11 shows that the size of MCDS reduces when the network density increases. A low density for networks of size more than 110 nodes leads to a dramatic increase in the computation time.

## Conclusions

Experimental identification of a set of key regulatory genes among large sets of genes is very time-consuming and costly. Therefore, computational methods such as the ones presented here are helpful to condense and shape a list of candidate genes to more promising candidates before planning and starting expensive experimental work. Such follow-up works could e.g. validate the regulatory roles of these genes by siRNA knockdown experiments, by over-expressing genes e.g. under the control of the highly inducible GAL1 promoter in yeast, or by CRISPR-type genome editing of promoter sequences containing TF binding sites. We presented three novel approaches (ILP formulation for the directed form of MDS, ILP formulation for the directed form of MCDS and heuristic algorithm for the directed form of MCDS) to identify driver genes and master regulatory genes responsible for a particular cellular identity. In the notion of network controllability, MDSs and MCDSs of biologicals networks are likely enriched in key regulatory genes. The results of these optimization problems can thus aid in pruning the network to the potentially more important nodes. We applied our method to the established GRNs of *E. coli* and *S. cerevisiae* and also to a pluripotency network of mouse ESC. The characteristics of these methods appear to be well suited, on the one hand, to the topology of approximately scale-free biological networks that contain a small number of high degree hub nodes and, on the other hand, to the observed tendency of these hubs to interact with each other. We showed that the networks can be controlled by a fairly small set of dominating TFs. A notable number of known master regulatory genes are detected in the connected dominating set of the components.

The number of driver genes obtained by the directed form of MDS and MCDS depends on the connectivity of the network. Networks with low connectivity yield a higher number of driver genes compared to networks with higher connectivity. The application of the MCDS method to modules of a regulatory network for a breast cancer network identified 70 key driver genes that could possibly drive the tumorigenesis process. Twenty of them are already known targets of available cancer drugs. The remaining dominating genes may be suitable candidates as news drug targets that may warrant further experimental validation.

## Additional files

Additional file 1 MDS. This file includes the implementation of ILP formulation for MDS problem using glpk solver. (SAGE 1 kb)

Additional file 2 MCDS. This file includes the implementation of ILP formulation for MCDS problem using glpk solver. (SAGE 4 kb)

Additional file 3 User guide. This guide contains instructions for users to use the MDS and MCDS programs to find the optimal solution in a directed network. It also includes two GRNs from breast cancer network modules which can be used as input networks for ILP programs. (PDF 45 kb)

Additional file 4 Data.This folder contains GRN files for *E. coli*, *S. cerevisiae*, pluripotency network of mouse ESC and breast cancer network modules. (ZIP 278 kb)

Additional file 5 Supplementary. This file includes the supplementary figures and tables mentioned in the paper. (PDF 274 kb)

## Abbreviations

TF: Transcription factor
GRN: Gene regulatory network
ESC: Embryonic stem cell
MDS: Minimum dominating set
MCDS: Minimum connected dominating set
ILP: Integer linear programming
SCC: Strongly connected component
LCC: Largest connected component
BFS: Breadth first search
LSCC: Largest strongly connected component
BH: Benjamini & Hochberg
GO: Gene ontology
BP: Biological process
KS: Kolmogorov-Smirnov
coli: Escherichia coli
YPA: Yeast promoter atlas
