# Supplementary material for: Identification of key player genes in gene regulatory networks
Source: BMC Syst Biol. 2016 Sep 6;10(1):88. doi: 10.1186/s12918-016-0329-5 (PMC5011974; doi:10.1186/s12918-016-0329-5)
Supplement: Additional file 3 — User guide. This guide contains instructions for users to use the MDS and MCDS programs to find the optimal solution in a directed network. It also includes two GRNs from breast cancer network modules which can be used as input networks for ILP programs. (PDF 45 kb) [file 12918_2016_329_MOESM3_ESM.pdf]

User Guide

Maryam Nazarieh

August 9, 2016

1 Overview

This guide contains instructions for users how to use the provided tools, the minimum dominating set (MDS) and minimum connected dominating set (MCDS) in a directed network. The ILP formulations were written in the Sage software system and use the glpk solver to find the solutions. Please note that the ILP-based implementations have additional requirements that are explained in section 4.

2 MDS

The ILP formulation for the directed form of MDS finds a minimum number of dominators in the directed graph. The program is applied to the full network to find the optimal solution for the MDS problem. The program takes two arguments (input file, output file). The first argument is a tab-delimited file as an input network and the second argument is the output text file to which the results are saved in the user-specified path. The program can be executed in the terminal as follows:

```
./sage MDS_direct.sage [input_file] [output_file]
```

Suppose we have a network named black.csv, the following command outputs the MDS result for the mentioned input network.

.

```
./sage MDS_direct.sage black.csv mds_result.txt
```

3 MCDS

To find the optimal solution for the MCDS problem, we considered two types of components in the network. The largest connected component underly-

ing undirected graph (LCC) and the largest strongly connected component (LSCC). No feasible solution for MCDS exists in graphs that are not connected.

The ILP formulation for the directed form of MCDS finds a set of minimum connected dominators in the directed graph. The program takes three arguments (component type, input file, output file). The first argument determines the type of component which can be either LCC or LSCC. The component type needs to be specified explicitly in upper case or lower case as given in the example. The other two arguments are a tab-delimited file as input network as in section 2 and the output text file to which the results are saved in the user-specified path. The program can be executed in the terminal as follows:

```
./sage MCDS_direct.sage [Component] [input_file] [output_file]
```

Suppose we have a network named black.csv, the following command outputs the MCDS result for the mentioned input network.

```
./sage MCDS_direct.sage LCC black.csv mcds_result.txt
```

4 System requirements

The ILP formulations were written in the Sage software system based on the glpk solver. The Sage software system can be downloaded from the website: `http://www.sagemath.org`.
To learn how to download and install the glpk solver, you can read the corresponding document available at `http://kam.mff.cuni.cz/~elias/glpk.pdf`.
