# Supplementary material for: Identification of key player genes in gene regulatory networks
Source: BMC Syst Biol. 2016 Sep 6;10(1):88. doi: 10.1186/s12918-016-0329-5 (PMC5011974; doi:10.1186/s12918-016-0329-5)
Supplement: Additional file 5 — Supplementary. This file includes the supplementary figures and tables mentioned in the paper. (PDF 274 kb) [file 12918_2016_329_MOESM5_ESM.pdf]

Figures

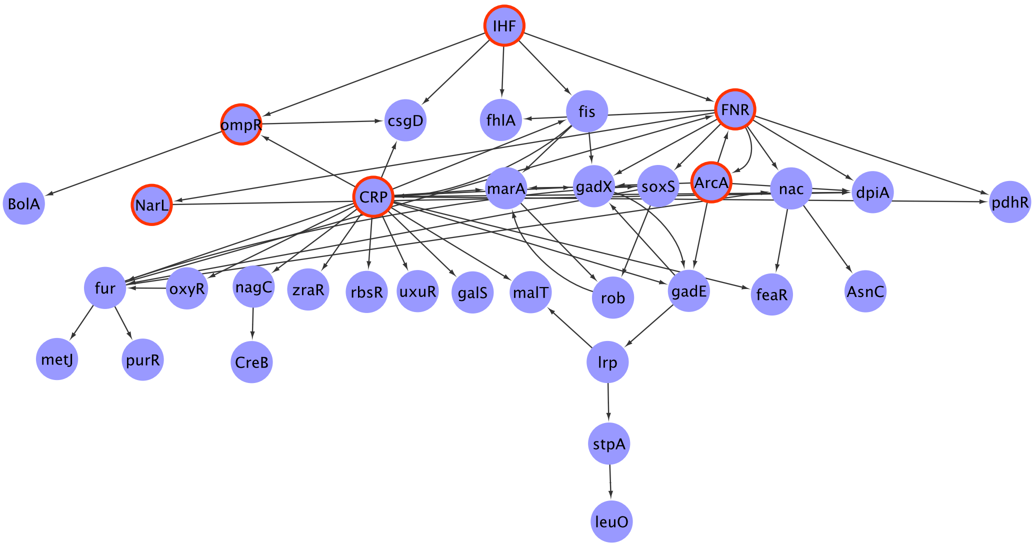

Figure S1: **Connectivity among the genes in the connected dominating set of the LCC of the *E.coli* GRN.** In this component, TFs construct the set of dominators and connectors. The red circle borders mark the master regulatory genes identified as global regulators in [32].

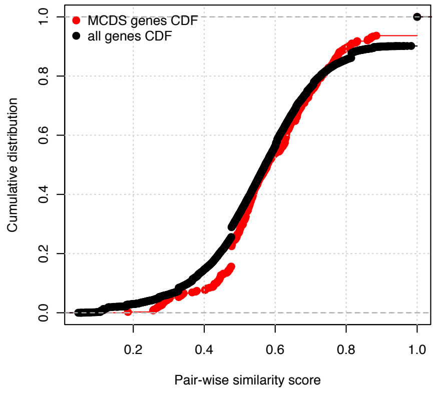

Figure S2: **Cumulative distribution of the functional similarity scores between pairs of MCDS nodes of the mouse pluripotency network (in red) against the similarity between all pairs of genes in the pluripotency network (in black).**

Tables

Table S1: 34 MCDS genes of the *E.coli* LCC based on RegulonDB. 'D' stands for dominating node, 'C' for connecting node. Only the largest component of the network with 1198 genes was analyzed here.

Gene	Role	no. Target genes
feaR	C	2
BolA	D	2
zraR	C	3
AsnC	D	4
CreB	D	5
stpA	C	7
uxuR	C	8
rbsR	C	9
galS	C	10
malT	C	10
dpiA	C	11
metJ	D	15
ompR	C	17
leuO	D	20
nac	C	21
csgD	C	23
rob	D	26
gadX	C	28
gadE	C	28
fhlA	C	30
purR	D	31
oxyR	C	33
nagC	C	36
marA	C	38
soxS	C	40
pdhR	C	42
lrp	D	105
narL	C	121
fur	C	129
ArcA	C	173
IHF	D	219
fis	C	227
fnr	D	296
CRP	D	497

Table S2: Enriched GO terms (top) and KEGG pathways with adjusted p-values < 0.05 for the 34 genes in the MCDS for the *E.coli* GRN. p-values were adjusted for multiple testing using the BH procedure.

Enriched terms	count	*adj. p-values*
GO:0006350 ∼ transcription	32	4.845E-28
GO:0045449 ∼ regulation of transcription	33	4.043E-26
GO:0051171 ∼ regulation of nitrogen compound metabolic process	33	3.204E-26
GO:0019219 ∼ regulation of nucleobase, nucleoside, nucleotide and nucleic acid metabolic process	33	3.204E-26
GO:0010556 ∼ regulation of macromolecule biosynthetic process	33	3.108E-26
GO:0009889 ∼ regulation of biosynthetic process	33	3.108E-26
GO:0031326 ∼ regulation of cellular biosynthetic process	33	3.108E-26
GO:0080090 ∼ regulation of primary metabolic process	33	2.843E-26
GO:0031323 ∼ regulation of cellular metabolic process	33	2.610E-26
GO:0006355 ∼ regulation of transcription, DNA-dependent	32	3.767E-26
GO:0051252 ∼ regulation of RNA metabolic process	32	3.572E-26
GO:0010468 ∼ regulation of gene expression	33	6.725E-26
GO:0060255 ∼ regulation of macromolecule metabolic process	33	7.117E-26
GO:0019222 ∼ regulation of metabolic process	33	7.779E-26
GO:0010467 ∼ gene expression	32	2.898E-25
GO:0050794 ∼ regulation of cellular process	33	1.280E-24
GO:0050789 ∼ regulation of biological process	33	6.833E-24
GO:0065007 ∼ biological regulation	33	1.549E-23
GO:0034645 ∼ cellular macromolecule biosynthetic process	32	1.114E-22
GO:0009059 ∼ macromolecule biosynthetic process	32	2.139E-22
GO:0044249 ∼ cellular biosynthetic process	32	3.984E-17
GO:0009058 ∼ biosynthetic process	32	3.175E-16
GO:0044260 ∼ cellular macromolecule metabolic process	33	2.886E-14
GO:0006139 ∼ nucleobase, nucleoside, nucleotide and nucleic acid metabolic process	32	7.960E-14
GO:0034641 ∼ cellular nitrogen compound metabolic process	33	4.637E-13
GO:0043170 ∼ macromolecule metabolic process	33	1.015E-12
GO:0006807 ∼ nitrogen compound metabolic process	33	1.708E-12
GO:0044238 ∼ primary metabolic process	34	7.490E-10
GO:0044237 ∼ cellular metabolic process	34	1.056E-9
GO:0009987 ∼ cellular process	34	2.724E-7
GO:0000160 ∼ two-component signal transduction system (phosphorelay)	10	1.913E-6
GO:0008152 ∼ metabolic process	34	4.584E-5
ecd02020:Two-component system	6	3.558E-7
ect02020:Two-component system	6	1.853E-7
ecr02020:Two-component system	6	1.853E-7
ecg02020:Two-component system	6	1.340E-7
eck02020:Two-component system	6	1.340E-7
ecq02020:Two-component system	6	1.088E-7
eum02020:Two-component system	6	9.060E-8
ecz02020:Two-component system	6	9.060E-8
ecf02020:Two-component system	6	9.060E-8
ecx02020:Two-component system	6	8.481E-8
ecj02020:Two-component system	6	8.481E-8
eco02020:Two-component system	6	7.553E-8
ecm02020:Two-component system	6	7.127E-8
ecv02020:Two-component system	6	6.576E-8
ecw02020:Two-component system	5	5.241E-6
eci02020:Two-component system	5	5.952E-6
ece02020:Two-component system	5	5.627E-6
ecc02020:Two-component system	5	5.688E-6

Table S3: 12 dominators in the identified MDS for the cell-cycle specific GRN of *S. cerevisiae.*

Genes
FKH1, GCR1, ORC1, YOX1, PHD1, ACE2, STB1, SWI5, STB5, SWI4, TEC1, RAP1

Table S4: 14 TFs and 3 target genes in the identified MCDS for the cell-cycle specific GRN of *S. cerevisiae.* 'C' and 'D' stand for the roles of connector and dominating nodes.

Gene	Role	no. Target genes
PMA2	C	0
TID3	C	0
CAR1	C	0
STP4	C	1
SRD1	C	3
GCR1	D	11
STB5	D	12
PHD1	D	18
YOX1	C	29
ACE2	C	31
RAP1	D	35
SWI5	C	43
SWI4	D	46
TEC1	C	47
ORC1	D	61
STB1	D	62
FKH1	D	96

Table S5: Enriched GO terms (top) and KEGG pathway (bottom line) with adjusted p-values < 0.05 for the 17 genes in the MCDS for the cell-cycle specific GRN of *S. cerevisiae*. p-values were adjusted for multiple testing using the BH procedure.

Enriched terms	count	*adj. P-values*
GO:0006355 ∼ regulation of transcription, DNA-dependent	12	2.952E-6
GO:0051252 ∼ regulation of RNA metabolic process	12	1.813E-6
GO:0045449 ∼ regulation of transcription	12	4.110E-5
GO:0019219 ∼ regulation of nucleobase, nucleoside, nucleotide and nucleic acid metabolic process	12	7.514E-5
GO:0051171 ∼ regulation of nitrogen compound metabolic process	12	6.098E-5
GO:0034645 ∼ cellular macromolecule biosynthetic process	15	1.015E-4
GO:0009059 ∼ macromolecule biosynthetic process	15	9.269E-5
GO:0010468 ∼ regulation of gene expression	12	2.122E-4
GO:0010556 ∼ regulation of macromolecule biosynthetic process	12	2.201E-4
GO:0006357 ∼ regulation of transcription from RNA polymerase II promoter	8	2.219E-4
GO:0010467 ∼ gene expression	15	2.078E-4
GO:0031326 ∼ regulation of cellular biosynthetic process	12	1.921E-4
GO:0009889 ∼ regulation of biosynthetic process	12	1.878E-4
GO:0006350 ∼ transcription	10	2.838E-4
GO:0060255 ∼ regulation of macromolecule metabolic process	12	4.468E-4
GO:0031323 ∼ regulation of cellular metabolic process	12	4.545E-4
GO:0080090 ∼ regulation of primary metabolic process	12	4.592E-4
GO:0019222 ∼ regulation of metabolic process	12	8.004E-4
GO:0048522 ∼ positive regulation of cellular process	7	8.463E-4
GO:0044249 ∼ cellular biosynthetic process	15	0.001
GO:0048518 ∼ positive regulation of biological process	7	0.001
GO:0009058 ∼ biosynthetic process	15	0.001
GO:0010628 ∼ positive regulation of gene expression	6	0.001
GO:0045941 ∼ positive regulation of transcription	6	0.001
GO:0051173 ∼ positive regulation of nitrogen compound metabolic process	6	0.002
GO:0045935 ∼ positive regulation of nucleobase, nucleoside, nucleotide and nucleic acid metabolic process	6	0.002
GO:0010557 ∼ positive regulation of macromolecule biosynthetic process 6 0.002		
GO:0009891 ∼ positive regulation of biosynthetic process	6	0.002
GO:0031328 ∼ positive regulation of cellular biosynthetic process	6	0.002
GO:0051329 ∼ interphase of mitotic cell cycle	5	0.002
GO:0051325 ∼ interphase	5	0.003
GO:0010604 ∼ positive regulation of macromolecule metabolic process	6	0.003
GO:0009893 ∼ positive regulation of metabolic process	6	0.003
GO:0031325 ∼ positive regulation of cellular metabolic process	6	0.003
GO:0034641 ∼ cellular nitrogen compound metabolic process	14	0.004
GO:0050794 ∼ regulation of cellular process	12	0.005
GO:0006807 ∼ nitrogen compound metabolic process	14	0.005
GO:0006139 ∼ nucleobase, nucleoside, nucleotide and nucleic acid metabolic process	13	0.006
GO:0044260 ∼ cellular macromolecule metabolic process	16	0.006
GO:0065007 ∼ biological regulation	13	0.008
GO:0043170 ∼ macromolecule metabolic process	16	0.008
GO:0050789 ∼ regulation of biological process	12	0.010
GO:0000278 ∼ mitotic cell cycle	6	0.014
sce04111:Cell cycle	4	0.051

Table S6: 29 MCDS genes of the mouse ESC pluripotency network identified in the largest SCC.

Gene	Role	out_degree
Wnt5a	C	1
Dvl1	D	1
Map2k1	D	1
Raf1	C	1
Dkk1	C	1
Smad3	D	1
Nedd4l	D	2
Akt1	C	2
Fgfr1	D	2
Grb2	D	2
Ptpn11	C	2
I tgb1	C	2
Tcl1	D	2
Mapk3	D	3
Lifr	D	3
Notch1	D	3
Hras1	D	3
Cdk2ap1	C	3
Cdx2	C	4
Gsk3b	D	5
Smad1	D	8
Sall4	C	9
Tcf3	D	12
Esrrb	D	14
Nr5a2	D	20
Stat3	D	20
Sox2	C	30
Nanog	C	46
Pou5f1	D	82

Table S7: Enriched GO terms and KEGG pathways with adjusted p-values < 0.05 for the 29 genes in the MCDS for the mouse pluripotency network. p-values were adjusted for multiple testing using the BH procedure.

Enriched terms	count	*adj. p-values*
GO:0009653 anatomical structure morphogenesis	19	5.814E-11
GO:0009790 embryonic development	16	9.890E-11
GO:0048513 organ development	21	7.347E-11
GO:0048731 system development	22	1.464E-10
GO:0032502 developmental process	24	2.106E-10
GO:0048519 negative regulation of biological process	19	1.957E-10
GO:0030154 cell differentiation	20	1.889E-10
GO:0048856 anatomical structure development	22	2.854E-10
GO:0048869 cellular developmental process	20	3.122E-10
GO:0048523 negative regulation of cellular process	18	3.431E-10
GO:0048518 positive regulation of biological process	19	8.095E-10
GO:0048522 positive regulation of cellular process	18	1.479E-9
GO:0048598 embryonic morphogenesis	12	2.233E-9
GO:0001701 in utero embryonic development	11	2.790E-9
GO:0007275 multicellular organismal development	22	2.957E-9
GO:0050793 regulation of developmental process	13	1.355E-8
GO:0051093 negative regulation of developmental process	10	1.501E-8
GO:0019827 stem cell maintenance	6	2.525E-8
GO:0048468 cell development	13	2.638E-8
GO:0048864 stem cell development	6	2.900E-8
GO:0009888 tissue development	13	3.913E-8
GO:0048646 anatomical structure formation involved in morphogenesis	11	3.778E-8
GO:0045596 negative regulation of cell differentiation	9	5.834E-8
GO:0045595 regulation of cell differentiation	11	5.952E-8
GO:0042127 regulation of cell proliferation	12	8.561E-8
GO:0048863 stem cell differentiation	6	1.114E-7
GO:0043009 chordate embryonic development	11	1.221E-7
GO:0009792 embryonic development ending in birth or egg hatching	11	1.289E-7
GO:0045165 cell fate commitment	8	3.238E-7
GO:0050789 regulation of biological process	28	3.465E-7
GO:0007167 enzyme linked receptor protein signaling pathway	9	1.042E-6
GO:0065007 biological regulation	28	1.328E-6
GO:0050794 regulation of cellular process	27	1.525E-6
GO:0009887 organ morphogenesis	11	2.550E-6
GO:0006357 regulation of transcription from RNA polymerase II promoter	11	3.481E-6
GO:0007243 protein kinase cascade	8	6.748E-6
GO:0045944 positive regulation of transcription from RNA polymerase II promoter	9	7.026E-6
GO:0045893 positive regulation of transcription, DNA-dependent	9	2.135E-5
GO:0008284 positive regulation of cell proliferation	8	2.171E-5
GO:0051254 positive regulation of RNA metabolic process	9	2.141E-5
GO:0032501 multicellular organismal process	22	3.182E-5
GO:0007169 transmembrane receptor protein tyrosine kinase signaling pathway	7	3.416E-5
GO:0010604 positive regulation of macromolecule metabolic process	10	4.234E-5
GO:0031325 positive regulation of cellular metabolic process	10	4.771E-5
GO:0045941 positive regulation of transcription	9	4.873E-5
GO:0010628 positive regulation of gene expression	9	5.828E-5
GO:0044260 cellular macromolecule metabolic process	22	6.809E-5
GO:0009893 positive regulation of metabolic process	10	7.095E-5
GO:0045935 positive regulation of nucleobase, nucleoside, nucleotide and nucleic acid metabolic process	9	7.588E-5
GO:0007242 intracellular signaling cascade	11	9.113E-5
GO:0051173 positive regulation of nitrogen compound metabolic process	9	9.160E-5
GO:0010557 positive regulation of macromolecule biosynthetic process	9	9.500E-5
GO:0048568 embryonic organ development	7	1.007E-4
GO:0031328 positive regulation of cellular biosynthetic process	9	1.234E-4
GO:0009891 positive regulation of biosynthetic process	9	1.294E-4
GO:0051239 regulation of multicellular organismal process	10	1.755E-4
GO:0000003 reproduction	9	2.858E-4

Enriched terms	count	*adj. p-values*
GO:0007399 nervous system development	10	3.578E-4
GO:0001890 placenta development	5	3.527E-4
GO:0019222 regulation of metabolic process	17	3.675E-4
GO:0043170 macromolecule metabolic process	22	4.118E-4
GO:0040007 growth	6	4.679E-4
GO:0030326 embryonic limb morphogenesis	5	5.073E-4
GO:0035113 embryonic appendage morphogenesis	5	5.073E-4
GO:0048589 developmental growth	5	5.629E-4
GO:0001707 mesoderm formation	4	6.255E-4
GO:0006355 regulation of transcription, DNA-dependent	12	7.191E-4
GO:0051094 positive regulation of developmental process	6	7.089E-4
GO:0048332 mesoderm morphogenesis	4	7.045E-4
GO:0001710 mesodermal cell fate commitment	3	7.035E-4
GO:0051252 regulation of RNA metabolic process	12	7.834E-4
GO:0010468 regulation of gene expression	15	7.754E-4
GO:0031323 regulation of cellular metabolic process	16	7.767E-4
GO:0001704 formation of primary germ layer	4	7.666E-4
GO:0000165 MAPKKK cascade	5	8.128E-4
GO:0035107 appendage morphogenesis	5	8.297E-4
GO:0035108 limb morphogenesis	5	8.297E-4
GO:0048333 mesodermal cell differentiation	3	8.931E-4
GO:0000122 negative regulation of transcription from RNA polymerase II promoter	6	8.864E-4
GO:0048736 appendage development	5	9.114E-4
GO:0060173 limb development	5	9.114E-4
GO:0010646 regulation of cell communication	9	9.341E-4
GO:0007166 cell surface receptor linked signal transduction	15	9.336E-4
GO:0048729 tissue morphogenesis	6	9.703E-4
GO:0030182 neuron differentiation	7	0.001
GO:0022414 reproductive process	8	0.001
GO:0006468 protein amino acid phosphorylation	8	0.001
GO:0060255 regulation of macromolecule metabolic process	15	0.002
GO:0001708 cell fate specification	4	0.002
GO:0007498 mesoderm development	4	0.002
GO:0007420 brain development	6	0.002
GO:0009966 regulation of signal transduction	8	0.002
GO:0042221 response to chemical stimulus	9	0.002
GO:0001892 embryonic placenta development	4	0.002
GO:0010556 regulation of macromolecule biosynthetic process	14	0.002
GO:0044237 cellular metabolic process	22	0.002
GO:0045892 negative regulation of transcription, DNA-dependent	6	0.002
GO:0051253 negative regulation of RNA metabolic process	6	0.002
GO:0006350 transcription	12	0.002
GO:0007439 ectodermal gut development	3	0.003
GO:0048567 ectodermal gut morphogenesis	3	0.003
GO:0031326 regulation of cellular biosynthetic process	14	0.003
GO:0048699 generation of neurons	7	0.003
GO:0009889 regulation of biosynthetic process	14	0.003
GO:0045597 positive regulation of cell differentiation	5	0.003
GO:0016310 phosphorylation	8	0.003
GO:0007369 gastrulation	4	0.003
GO:0001829 trophectodermal cell differentiation	3	0.003
GO:0006464 protein modification process	10	0.003
GO:0045995 regulation of embryonic development	3	0.004
GO:0022008 neurogenesis	7	0.004
GO:0045449 regulation of transcription	13	0.004
GO:0048732 gland development	5	0.004
GO:0043412 biopolymer modification	10	0.005
GO:0007417 central nervous system development	6	0.005

Enriched terms	count	*adj. p-values*
GO:0016481 negative regulation of transcription	6	0.005
GO:0043687 post-translational protein modification	9	0.005
GO:0009987 cellular process	27	0.005
GO:0051726 regulation of cell cycle	5	0.005
GO:0080090 regulation of primary metabolic process	14	0.005
GO:0001825 blastocyst formation	3	0.006
GO:0019219 regulation of nucleobase, nucleoside, nucleotide and nucleic acid metabolic process	13	0.006
GO:0045934 negative regulation of nucleobase, nucleoside, nucleotide and nucleic acid metabolic process	6	0.007
GO:0051171 regulation of nitrogen compound metabolic process	13	0.007
GO:0051172 negative regulation of nitrogen compound metabolic process	6	0.007
GO:0034645 cellular macromolecule biosynthetic process	13	0.007
GO:0010629 negative regulation of gene expression	6	0.007
GO:0009059 macromolecule biosynthetic process	13	0.007
GO:0006793 phosphorus metabolic process	8	0.008
GO:0006796 phosphate metabolic process	8	0.008
GO:0043066 negative regulation of apoptosis	5	0.008
GO:0045667 regulation of osteoblast differentiation	3	0.008
GO:0048547 gut morphogenesis	3	0.008
GO:0010558 negative regulation of macromolecule biosynthetic process	6	0.008
GO:0001568 blood vessel development	5	0.008
GO:0043069 negative regulation of programmed cell death	5	0.008
GO:0060548 negative regulation of cell death	5	0.008
GO:0008283 cell proliferation	5	0.008
GO:0001944 vasculature development	5	0.008
GO:0031327 negative regulation of cellular biosynthetic process	6	0.009
GO:0009890 negative regulation of biosynthetic process	6	0.009
GO:0031667 response to nutrient levels	4	0.009
GO:0007492 endoderm development	3	0.009
GO:0032526 response to retinoic acid	3	0.009
GO:0033189 response to vitamin A	3	0.010
GO:0003006 reproductive developmental process	5	0.010
GO:0033273 response to vitamin	3	0.011
GO:0060541 respiratory system development	4	0.011
GO:0001763 morphogenesis of a branching structure	4	0.011
GO:0048546 digestive tract morphogenesis	3	0.011
GO:0055123 digestive system development	3	0.012
GO:0016055 Wnt receptor signaling pathway	4	0.012
GO:0060711 labyrinthine layer development	3	0.012
GO:0009880 embryonic pattern specification	3	0.012
GO:0001501 skeletal system development	5	0.013
GO:0007389 pattern specification process	5	0.013
GO:0009991 response to extracellular stimulus	4	0.013
GO:0007398 ectoderm development	4	0.013
GO:0051049 regulation of transport	5	0.013
GO:0016043 cellular component organization	11	0.013
GO:0031324 negative regulation of cellular metabolic process	6	0.013
GO:0048565 gut development	3	0.015
GO:0010033 response to organic substance	6	0.015
GO:0010605 negative regulation of macromolecule metabolic process	6	0.015
GO:0044238 primary metabolic process	21	0.015
GO:0030030 cell projection organization	5	0.018
GO:0007165 signal transduction	13	0.018
GO:0009892 negative regulation of metabolic process	6	0.019
GO:0009725 response to hormone stimulus	4	0.022
GO:0030900 forebrain development	4	0.022
GO:0051716 cellular response to stimulus	6	0.022
GO:0050896 response to stimulus	12	0.024
GO:0032989 cellular component morphogenesis	5	0.024
GO:0030278 regulation of ossification	3	0.025
GO:0001824 blastocyst development	3	0.026
GO:0008152 metabolic process	22	0.026
GO:0009719 response to endogenous stimulus	4	0.028

Enriched terms	count	*adj. p-values*
GO:0032870 cellular response to hormone stimulus	3	0.029
GO:0007049 cell cycle	6	0.031
GO:0006950 response to stress	8	0.034
GO:0050678 regulation of epithelial cell proliferation	3	0.034
GO:0010467 gene expression	12	0.034
GO:0044249 cellular biosynthetic process	13	0.036
GO:0009605 response to external stimulus	6	0.037
GO:0032879 regulation of localization	5	0.038
GO:0048852 diencephalon morphogenesis	2	0.038
GO:0000904 cell morphogenesis involved in differentiation	4	0.039
GO:0031175 neuron projection development	4	0.042
GO:0051051 negative regulation of transport	3	0.042
GO:0035270 endocrine system development	3	0.042
GO:0006366 transcription from RNA polymerase II promoter	3	0.044
GO:0007507 heart development	4	0.044
GO:0009058 biosynthetic process	13	0.044
GO:0007584 response to nutrient	3	0.045
GO:0051216 cartilage development	3	0.047
mmu05200:Pathways in cancer	13	1.902E-9
mmu05220:Chronic myeloid leukemia	8	7.787E-8
mmu05210:Colorectal cancer	8	1.252E-7
mmu05215:Prostate cancer	8	1.296E-7
mmu05213:Endometrial cancer	7	1.327E-7
mmu05221:Acute myeloid leukemia	7	1.952E-7
mmu05211:Renal cell carcinoma	7	5.896E-7
mmu04722:Neurotrophin signaling pathway	8	8.510E-7
mmu04662:B cell receptor signaling pathway	7	1.030E-6
mmu04012:ErbB signaling pathway	7	1.537E-6
mmu04916:Melanogenesis	7	3.216E-6
mmu05223:Non-small cell lung cancer	6	3.483E-6
mmu04062:Chemokine signaling pathway	8	5.253E-6
mmu04660:T cell receptor signaling pathway	7	6.742E-6
mmu05214:Glioma	6	6.581E-6
mmu04510:Focal adhesion	8	7.542E-6
mmu05218:Melanoma	6	9.775E-6
mmu05212:Pancreatic cancer	6	9.901E-6
mmu04910:Insulin signaling pathway	7	1.245E-5
mmu04664:Fc epsilon RI signaling pathway	6	1.702E-5
mmu04650:Natural killer cell mediated cytotoxicity	6	1.134E-4
mmu04320:Dorso-ventral axis formation	4	1.346E-4
mmu04370:VEGF signaling pathway	5	2.677E-4
mmu04010:MAPK signaling pathway	7	4.049E-4
mmu04540:Gap junction	5	3.992E-4
mmu04912:GnRH signaling pathway	5	6.119E-4
mmu05219:Bladder cancer	4	7.840E-4
mmu04810:Regulation of actin cytoskeleton	6	0.001
mmu04310:Wnt signaling pathway	5	0.002
mmu04630:Jak-STAT signaling pathway	5	0.002
mmu04720:Long-term potentiation	4	0.003
mmu04730:Long-term depression	4	0.003
mmu04914:Progesterone-mediated oocyte maturation	4	0.005
mmu04666:Fc gamma R-mediated phagocytosis	4	0.007
mmu05216:Thyroid cancer	3	0.007
mmu05020:Prion diseases	3	0.010
mmu04360:Axon guidance	4	0.015
mmu05217:Basal cell carcinoma	3	0.023
mmu04920:Adipocytokine signaling pathway	3	0.033
mmu04520:Adherens junction	3	0.041
mmu04350:TGF-beta signaling pathway	3	0.051

Table S8: Runtime to determine an optimal solution for individual modules in the BC network with differing number of nodes and edges. Listed is also the resulting component density. All computations were conducted on a single threaded Intel XEON machine running at 2.2 Ghz. The module (grey) could not be solved in appropriate time using ILP (not even after a month)

Module	Nodes	Edges	Density	Running Time (s)
black	41	233	0.13	1.91
brown	195	18843	0.49	52.71
green	110	595	0.04	94.60
magenta	26	123	0.18	1.91
pink	30	149	0.16	1.82
red	93	473	0.05	3.02
yellow	132	663	0.03	3499.55
blue	247	30239	0.49	14287.19
turquoise	295	43213	0.49	266.18
grey	148	723	0.03	NA

Table S9: Identified genes in the MCDS (heuristic approach) for 10 modules of the breast cancer network. The genes, whose protein products are known to be targeted by drugs, are marked in bold.

Method	Module	Network Size	Result size	Key driver genes
MCDS	black	41	8	ZNF254, SEC24B, ZNF681, ZZZ3, WNT5B, **CEP350**, ZNF426, ZNF137
MCDS	blue	247	3	FAM54A,**ACAN**, PDGFA
MCDS	brown	195	1	**AATK**
MCDS	green	110	29	GCDH, HDAC10, FLII, PIGQ, CXXC1, CNDP2, DPP7, **OS9**, MAGOH, **AKT1**, E4F1, UTP14A, SH3GLB2, ATPBD4, C9orf7, **AP1B1**, TIMM44, WDR13, USF2, USF1, CDK9, UCK1, **CDC34**, PQLC1, KIAA0664, **CDC37**, C7orf27, **CDK10**, LTBR
MCDS	grey	148	38	FAM59A, FBN2, COLEC10, FRMPD1, IL5RA, RORA, GEFT, RNF2, **CACNA1H**, CLGN, **CAMK2N1**, CEP72, **CA6**, PRND, ZC3H14, OR7A17, PRPH, **BRD1**, TAF15, ZRANB2, ZNF480, **ANXA13**, UPF3A , SNCAIP, HPCAL4, MR1, POU4F2, SYT6, DHDDS, CXorf26, GRHPR, PHF16, CNTN4, LHX4, **CCDC130**, PPARA , **ABCG4**, SPRR1B
MCDS	magenta	26	7	VPS72, **BGLAP**, SESN2, TAF7, MED26, ILF2, **ATF6**
MCDS	pink	30	6	ZNF706 , TCEB1, CHRAC1, DHX35, ZNF250, TMEM70
MCDS	red	93	22	TGIF1, ZNF485, ZNF691, **TGFB1**, DHX8, USP21, PHF20, GTF3A, FHL3, RPS3A , JOSD2 , **ATP1B1**, SUMF2, EPN3, HTR6, PPP4C, **CCDC9**, **MYC**, UBAP1, PCGF1, C6orf134, **TP53**
MCDS	tur quoise	295	1	**ABHD10**
MCDS	yellow	132	26	SPI1, TRAF3IP3, HDAC11, HTRA4, CXCR4, IL2RG, ETS1, FUT4, FAM129C, FAM124B , **CASP10**, RASSF5, PHACTR2, TSPAN2, PAG1, SLAMF1, SLC31A2, **DAPK1**, TNFRSF9, **NFKB1**, FLI1, GZMK, SLFN11, PRKD3, LCP2, DFNA5

Table S10: Enriched GO terms and KEGG pathways with adjusted p-values < 0.05 for the 141 genes in the aggregated MCDS for the modules of the breast cancer network. p-values were adjusted for multiple testing using the BH procedure.

Enriched terms	count	*adj. p-values*
GO:0009059~macromolecule biosynthetic process	43	0.001
GO:0010468~regulation of gene expression	43	0.001
GO:0006357~regulation of transcription from RNA polymerase II promoter	20	0.001
GO:0032774~RNA biosynthetic process	12	0.001
GO:0051254~positive regulation of RNA metabolic process	15	0.001
GO:0006350~transcription	37	0.001
GO:0010556~regulation of macromolecule biosynthetic process	43	0.001
GO:0032583~regulation of gene-specific transcription	9	0.001
GO:0009889~regulation of biosynthetic process	43	0.001
GO:0009891~positive regulation of biosynthetic process	18	0.001
GO:0045449~regulation of transcription	40	0.001
GO:0010467~gene expression	43	0.001
GO:0006351~transcription, DNA-dependent	12	0.001
GO:0045893~positive regulation of transcription, DNA-dependent	15	0.001
GO:0034645~cellular macromolecule biosynthetic process	43	0.001
GO:0031326~regulation of cellular biosynthetic process	43	0.001
GO:0051171~regulation of nitrogen compound metabolic process	42	0.002
GO:0019219~regulation of nucleobase, nucleoside, nucleotide and nucleic acid metabolic process	41	0.002
GO:0060255~regulation of macromolecule metabolic process	45	0.002
GO:0051173~positive regulation of nitrogen compound metabolic process	17	0.002
GO:0000122~negative regulation of transcription from RNA polymerase II promoter	11	0.002
GO:0019222~regulation of metabolic process	48	0.002
GO:0010557~positive regulation of macromolecule biosynthetic process	17	0.002
GO:0006139~nucleobase, nucleoside, nucleotide and nucleic acid metabolic process	46	0.002
GO:0043170~macromolecule metabolic process	65	0.003
GO:0034641~cellular nitrogen compound metabolic process	48	0.003
GO:0031323~regulation of cellular metabolic process	46	0.003
GO:0031328~positive regulation of cellular biosynthetic process	17	0.003
GO:0045935~positive regulation of nucleobase, nucleoside, nucleotide and nucleic acid metabolic process	16	0.003
GO:0010551~regulation of specific transcription from RNA polymerase II promoter	7	0.003
GO:0080090~regulation of primary metabolic process	44	0.004
GO:0045941~positive regulation of transcription	15	0.004
GO:0045944~positive regulation of transcription from RNA polymerase II promoter	12	0.004
GO:0044260~cellular macromolecule metabolic process	60	0.004
GO:0044249~cellular biosynthetic process	45	0.004
GO:0006807~nitrogen compound metabolic process	48	0.004
GO:0010628~positive regulation of gene expression	15	0.004
GO:0006355~regulation of transcription, DNA-dependent	29	0.005
GO:0051252~regulation of RNA metabolic process	29	0.006
GO:0009058~biosynthetic process	45	0.008
GO:0045892~negative regulation of transcription, DNA-dependent	11	0.013
GO:0006366~transcription from RNA polymerase II promoter	9	0.014
GO:0051253~negative regulation of RNA metabolic process	11	0.014
GO:0010629~negative regulation of gene expression	13	0.014
GO:0043193~positive regulation of gene-specific transcription	6	0.016
GO:0009893~positive regulation of metabolic process	18	0.018
GO:0016070~RNA metabolic process	18	0.021
GO:0031667~response to nutrient levels	8	0.021
GO:0010604~positive regulation of macromolecule metabolic process	17	0.022
GO:0016481~negative regulation of transcription	12	0.022
GO:0031325~positive regulation of cellular metabolic process	17	0.027
GO:0031327~negative regulation of cellular biosynthetic process	13	0.031
GO:0045595~regulation of cell differentiation	12	0.034
GO:0009890~negative regulation of biosynthetic process	13	0.036
GO:0009991~response to extracellular stimulus	8	0.037
GO:0019216~regulation of lipid metabolic process	6	0.040
GO:0045934~negative regulation of nucleobase, nucleoside, nucleotide and nucleic acid metabolic process	12	0.044
GO:0051172~negative regulation of nitrogen compound metabolic process	12	0.048
GO:0009892~negative regulation of metabolic process	15	0.055
hsa05200:Pathways in cancer	12	0.004

Table S11: Runtime to determine an optimal solution for generated directed random networks with differing number of nodes and edges. Listed is also the resulting component density. All computations were conducted on a single threaded Intel XEON machine running at 2.2 Ghz. The networks whose running times exceeded 2 days were discarded.

nodes	edges	density	mcds_size	mcds_time (s)
10	9	0.1	6	1.94
10	27	0.3	4	2.00
10	45	0.5	2	1.81
10	63	0.7	2	1.80
10	81	0.9	1	1.85
30	87	0.1	9	2.53
30	261	0.3	4	2.21
30	435	0.5	3	2.03
30	609	0.7	2	2.07
30	783	0.9	1	2.17
50	245	0.1	11	4.43
50	735	0.3	5	3.83
50	1225	0.5	3	8.77
50	1715	0.7	2	4.47
50	2205	0.9	1	3.03
70	483	0.1	11	5.69
70	1449	0.3	5	25.56
70	2415	0.5	3	19.89
70	3381	0.7	3	61.69
70	4347	0.9	2	43.32
90	801	0.1	12	35.16
90	2403	0.3	6	1467.69
90	4005	0.5	4	1022.77
90	5607	0.7	3	137.33
90	7209	0.9	2	42.01
110	1199	0.1	13	497.21
110	3597	0.3	5	1761.15
110	5995	0.5	4	3132.90
110	8393	0.7	3	455.06
110	10791	0.9	2	27.90
130	1677	0.1	13	4706.06
130	5031	0.3	6	8625.99
130	8385	0.5	4	9903.08
130	11739	0.7	3	959.93
130	15093	0.9	2	279.81
150	2235	0.1	13	5902.89
150	6705	0.3	6	21610.52
150	11175	0.5	4	24067.34
150	15645	0.7	3	1994.68
150	20115	0.9	2	810.58
170	2873	0.1	-	-
170	8619	0.3	-	-
170	14365	0.5	4	44398.62
170	20111	0.7	3	2867.04
170	25857	0.9	2	675.49
190	3591	0.1	-	-
190	10773	0.3	-	-
190	17955	0.5	4	85180.81
190	25137	0.7	3	4738.96
190	32319	0.9	2	854.05
